# Laparoscopic repair of sciatic hernia recognizing the ureterohypogastric nerve fascia and vesicohypogastric fascia: a case report

**DOI:** 10.1186/s40792-022-01362-4

**Published:** 2022-01-11

**Authors:** Mutsumi Fujimoto, Masashi Miguchi, Hiroshi Mitsuta, Satoshi Ikeda, Hideki Nakahara, Toshiyuki Itamoto

**Affiliations:** 1 Department of Gastroenterological Surgery, Hiroshima Prefectural Hospital, 1-5-54 Ujina-Kanda, Minami-ku, Hiroshima, 734-8530 Japan; 2 Department of Surgery, Otagawa Hospital, 1-21-25 Hesakasenzoku, Higashi-ku, Hiroshima, Japan; 3 Department of Gastroenterological and Transplant Surgery, Applied Life Sciences, Institute of Biomedical and Health Sciences, Hiroshima University, Hiroshima, Japan

**Keywords:** Laparoscopic surgery, Sciatic hernia, Suprapiriformis hernia

## Abstract

**Background:**

Sciatic hernias are rare pelvic floor hernias that occur through the sciatic foramen and often present as abdominal or pelvic pain, particularly in women. Historically, they were repaired using an open approach, with limited reports on their laparoscopic treatment.

**Case presentation:**

Here we present the case of an 85-year-old woman who had repeated abdominal pain and was referred to our hospital for sciatic hernia surgery after conservative treatment. We laparoscopically observed the deep pelvis and identified the right sciatic hernia. When an extraperitoneal space was dissected, an ureterohypogastric nerve fascia (UNF) and a vesicohypogastric fascia (VF) were identified. Moreover, the maneuver to mobilize the fasciae inside from the pelvic wall made it possible to separate the ureter and urinary bladder, which might have otherwise incarcerated in the hernia. We repaired the defect of the sciatic foramen with a mesh plug and patch. The patient had an uneventful recovery, and the absence of sciatic herniation recurrence was confirmed 1 year after surgery.

**Conclusion:**

A laparoscopic repair of a sciatic hernia could permit detailed non-invasive observations of the deep pelvis and be performed effectively by recognizing an UNF and a VF located near the sciatic foramen.

## Background

Sciatic hernia is one of the rarest forms of pelvic hernia, with only about 100 cases reported to date [1]. It accounts for ~ 0.01% of all abdominal hernias [1, 2] and is more prevalent in females. Sudden weight loss, liposarcoma, multiparity, and other types of hernias have been reported as predisposition factors for sciatic hernia [1, 2]. The sciatic foramen is the orifice of the sciatic hernia, which is rarely approached by general surgeons from the abdominal cavity, and there is a lack its anatomical understanding. However, the colorectal surgeons laparoscopically perform lateral lymph node dissection (LLND) for advanced rectal cancer and are anatomically familiar with the deep pelvis and sciatic foramen [3, 4]. Herein, we present a case of sciatic hernia diagnosed on computed tomography (CT) performed for bowel obstruction and successfully treated with a laparoscopic approach in reference to LLND.

## Case presentation

**Fig. 1:**
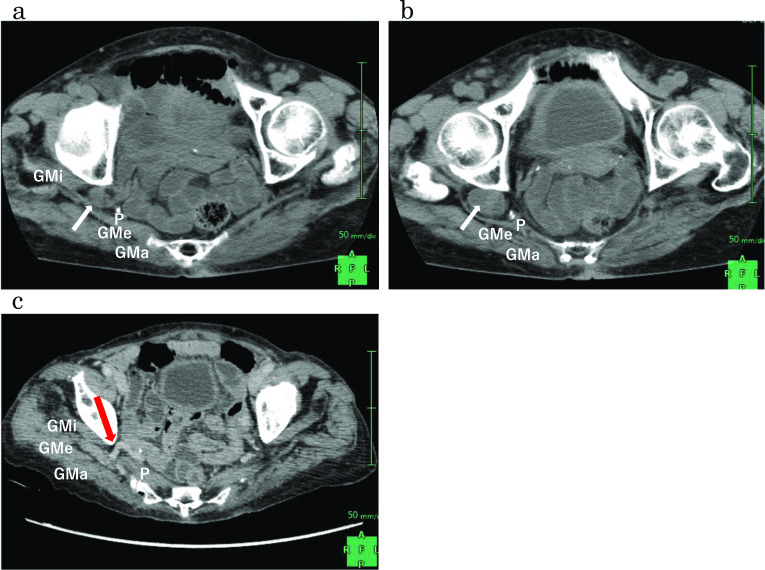
CT findings. **a**, **b** The small intestine herniated into the right greater sciatic foramen above the piriformis muscle (arrow). *P* piriformis muscle, *GMa* gluteus maximus muscle, *GMe* gluteus medius muscle, *GMi* gluteus minimus muscle. **c** A dilated superior gluteal vein was manifested when small bowel herniation was not observed in the right suprapiriform foramen (red arrow)

Four years ago, an 85-year-old woman experienced abdominal pain and consulted another hospital. She was diagnosed with bowel obstruction and hospitalized; her symptoms were relieved by conservative treatment. When the patient again experienced fever and abdominal pain, she consulted other hospitals and was hospitalized for bowel obstruction. CT demonstrated small intestine incarceration to the right sciatic hernia, which was thought to be the cause of the intestinal obstruction. After her symptoms were alleviated through conservative treatment, she was referred to our hospital. Physical examination revealed the following: height, 142 cm; weight, 38.5 kg (body mass index: 19.1). CT revealed that the small intestine had herniated into the sciatic foramen and the oral side of the small intestine had dilated (Fig. 1a, b). Furthermore, a right dilated superior gluteal vein without findings indicative of small bowel obstruction was observed (Fig. 1c).

**Fig. 2:**
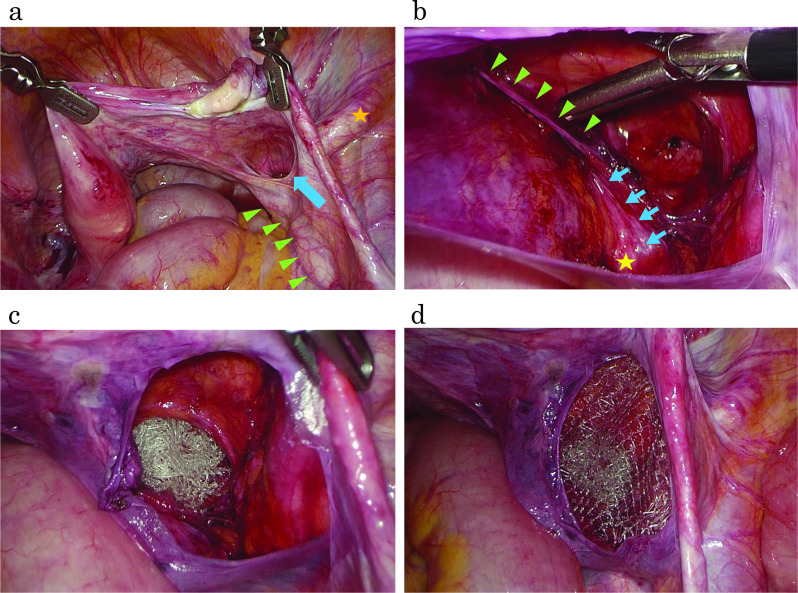
Intraoperative findings.** a** Laparoscopic view of the right sciatic hernia (arrow). The right ovary and adnexa were already retracted from the hernia defect using an FJ Clip (CHARMANT, Fukui, Japan). The arrowhead shows the right ureter, and the asterisk shows the right external iliac artery. **b** The arrow shows the ureterohypogastric nerve fascia, and the arrowhead shows the vesicohypogastric fascia, and the asterisk shows the right ureter. **c**,** d** The hernia defect was repaired with a Mesh Light Perfix Plug® (Bard Surgical, Cranston, RI), and an on-lay patch was placed over the plug

The patient was treated laparoscopically using three trocars and was positioned in a head-down position. The hernia was clearly evident in the suprapiriformis position of the greater sciatic foramen. Hernia orifice, 2.5 cm in diameter, was confirmed inside the right external iliac artery and outside the right ureter (Fig. 2a). The peritoneum was opened above the hernia orifice, and an extraperitoneal space was bluntly dissected. An ureterohypogastric nerve fascia (UNF) and a vesicohypogastric fascia (VF) were identified, and the fasciae were mobilized inside from the pelvic wall (Fig. 2b). This maneuver made it possible to separate the ureter and urinary bladder, which might have otherwise incarcerated in the hernia. As the tip of the hernia sac fell into the suprapiriform foramen, the hernia was diagnosed as a suprapiriform foramen hernia. The hernia defect was repaired with a Mesh Light Perfix Plug® (Bard Surgical, Cranston, RI). The plug was inserted into the hernia; an on-lay patch was placed over the plug (Fig. 2c, d). The overlying parietal peritoneum was closed with absorbable sutures. The patient had an uneventful recovery. One year postoperatively, hernia recurrence was not observed on CT.

## Discussion

**Fig. 3:**
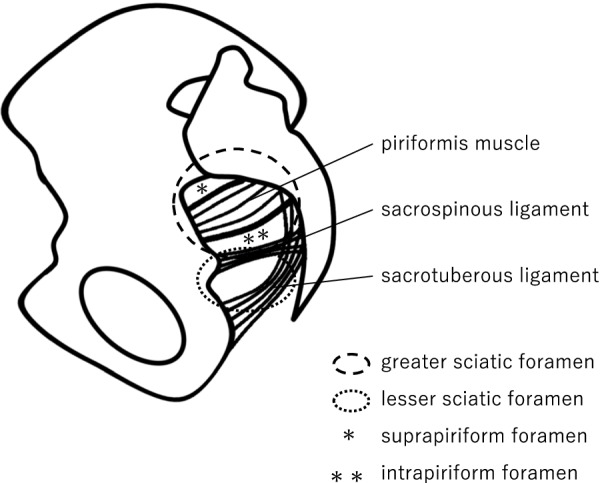
Diagram indicating the anatomy around the sciatic foramen. The greater sciatic foramen is formed by the great ischial notch, sacrospinous ligament, and sacrotuberous ligament. The greater sciatic foramen is further divided by the piriformis muscle into the suprapiriform and infrapiriform foramina. The lesser sciatic foramen is formed by the sacrospinous ligament, sacrotuberous ligament, and lesser sciatic notch. The single asterisk shows the suprapiriform foramen, and the double asterisk shows the infrapiriform foramen

Sciatic hernias are extremely rare; however, their reported incidence has increased in recent years due to the frequent use of radiological examination [1, 5]. Sciatic hernias may involve the ovary, small intestine, colon, greater omentum, ureter, and urinary bladder [2, 6]. The hernia can occur above (suprapiriformis hernia) or below the piriformis muscle (infrapiriformis hernia), or below the sacrospinous ligament (subspinous hernia) (Fig. 3). In suprapiriformis hernia, the hernial sac exits the pelvis above the piriformis muscle along the course of the superior gluteal vessels and nerve. On a multidetector CT, it has been reported to coexists with a dilated superior gluteal vein [5], consistent with the CT findings in the present case. The dilatation of the superior gluteal vein was evident without the incarceration of a suprapiriform foramen hernia. These findings would suggest the presence of an asymptomatic sciatic hernia. The majority of sciatic hernias are acquired conditions that occur due to chronically high intra-abdominal pressure. In such cases, advances age and pregnancy could lead to anatomic changes in the pelvic floor. In most cases, the patients are usually females with complaints of chronic and intermittent pelvic pain [7].

Regardless of the incarceration, the treatment of sciatic hernias is surgical reduction and repair due to the high risk of strangulation. Although a bilateral symptomatic sciatic hernia threated by robotic approach was reported [8], bilateral onset is very rare. It is not necessary to repair the contralateral side when the preoperative image and operative findings that suspects contralateral sciatic hernia are not observed. Sciatic hernia repair was historically performed through an open approach using a direct suture or a prosthetic material [1, 5, 9, 10]. However, the laparoscopic approach and repair with a plug or patch have been reported to show good feasibility and postoperative outcomes [7, 9], The excellent and detailed observation of this type of hernia with laparoscopy could enable precise diagnosis and facilitate its repair by allowing the recognition of all the anatomical elements [9]. Laparoscopic approach of sciatic hernias can result in a faster recovery, less pain, and better cosmetic results [2, 9, 11]. Even in the case of incarcerated sciatic hernia, laparoscopic approach is feasible if surgeons carefully perform the incarcerated hernia reduction so that intestinal perforation does not occur. However, when intestinal expansion resulting from bowel obstruction is extensive, an appropriate approach is open surgery, as it is expected that enough operation field is not secured under the laparoscope. The open approach should be used in cases with unstable vital signs due to septic shock, as the circulatory dynamics may become unstable due to the pneumoperitoneal effect of the laparoscopic approach. This laparoscopic technique requires the same skill set as laparoscopic inguinal hernia repair and, therefore, can be performed by almost any experienced laparoscopic hernia surgeon [9]. However, the opportunity when hernia surgeon experiences operation of the sciatic hernia is very rare, and there is not the report that formulated the operative procedure. On the other hand, the colorectal surgeons, perform LLND, we first identify a UNF, a VF, and a psoas muscle comprising the pelvic sidewall; we then dissect the bloc of lymph nodes located between these structures [3, 4]. In the present case, we first cut the retroperitoneum and mobilized the UNF inward. This procedure can return the hernia sac to the abdominal cavity and result in keeping away the ureter from the sciatic hernia orifice. Next, we identified and mobilized the VF to the inside direction. This procedure can keep away the urinary bladder connecting with the VF from the sciatic hernia orifice. As the space created by the hernia had some volume, plug placement into the space was effective for preventing hernia recurrence. The on-lay patch that was inserted in the dissected space outside the UNF and VF could prevent the ureter and urinary bladder from making contact with the plug’s sharp edge, which could cause injury.

## Conclusion

In conclusion, a laparoscopic repair of a sciatic hernia could permit detailed non-invasive observations. Furthermore, we recommend a laparoscopic repair recognizing the fasciae, such as a UNF and a VF.

## Data Availability

All data generated or analyzed during this study are included in this published article.

## Abbreviations

LLND: Lateral lymph node dissection
CT: Computed tomography
UNF: Ureterohypogastric nerve fascia
VF: Vesicohypogastric fascia
